# Upcycling Tunisian dental plaster waste into β-tricalcium phosphate: process optimization *via* Box–Behnken design and characterization

**DOI:** 10.1039/d6ra05107c

**Published:** 2026-08-14

**Authors:** Sondes Ftiti, Awatef Guidara, Sandra C. Cifuentes, Sophia Tsipas, Joaquín Rams, Basma Samet, Hassib Tounsi

**Affiliations:** a Laboratory of Advanced Materials (LR01ES26), Department of Materials and Environment, National Engineering School of Sfax, University of Sfax Tunisia sondes.ftiti@enis.tn; b Department of Applied Mathematics, Materials Science and Engineering and Electronic Technology, Universidad Rey Juan Carlos 28933 Móstoles Spain; c Department of Material Science and Engineering, Universidad Carlos III de Madrid 28911 Leganes Spain; d Institute for Research on Sustainable Technologies, Rey Juan Carlos University 28933 Móstoles Spain; e Department of Chemistry, Faculty of Science of Sfax, University of Sfax Tunisia

## Abstract

This research upcycles Tunisian dental plaster waste into β-tricalcium phosphate. The process effectively manages mineral by-products, preventing the accumulation of non-biodegradable dental gypsum waste. An effective Box–Behnken design and response surface methodology was used for optimizing the stoichiometric reaction between dental plaster, CaSO_4_·2H_2_O and phosphoric acid. The optimization focused on three critical variables: the solid/liquid (S/L) ratio, sintering temperature, and heating duration. Our results identified the ideal synthesis parameters as an S/L ratio of 3.8, a temperature of 1150 °C, and a 1 hour heating cycle. Comprehensive characterisation, comprising X-ray diffraction (XRD), Fourier-transform infrared spectroscopy (FTIR) and dilatometry, confirmed that the synthesised β-TCP was comparable to a commercial β-TCP. Scanning electron microscopy (SEM) imaging revealed a robust morphology consisting of fused blocks, mimicking bone architecture, and energy-dispersive X-ray spectrometry (EDX) analysis confirmed a Ca/P molar ratio of 1.48.

## Abstract 2

This research upcycles Tunisian dental plaster waste into β-tricalcium phosphate.

## Graphical abstract

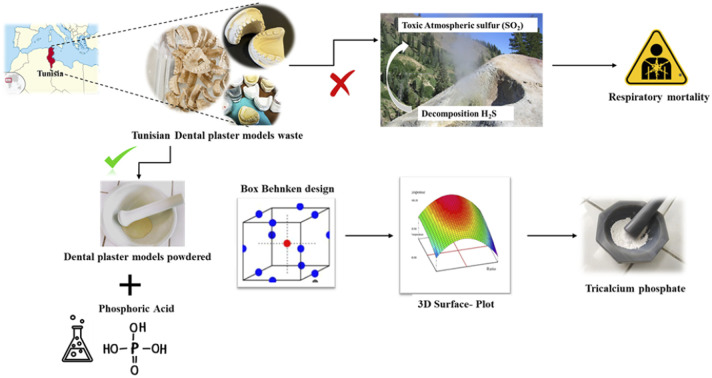

## Introduction

Dental laboratories generate a diverse waste stream. This waste is broadly categorized into infectious and non-infectious toxic residues. On the first hand, infectious waste, which often constitutes over 90% of a clinic's total solid output, comprises materials exposed to hematogenous or biological fluids, such as prostheses and orthodontic appliances.^1,2^ Due to this high volume, rigorous sterilization and specialized disposal protocols are essential.^2^ On the other hand, non-infectious waste is often biologically inert. However, it remains environmentally hazardous due to its chemical complexity. This category traditionally includes amalgam alloys, gypsum by-products, and acrylic resins. However, concerns have expanded to include nanoplastics released from 3D-printed polymers and aligners. These materials pose emerging health risks.^3^ A significant portion of this stream consists of gypsum by-products, such as dental stone, plaster of Paris (POP), and investment materials.^1,4^ Among gypsum by-products, dental plaster models (DPMs) are essential for diagnostics, treatment planning, and prosthetic fabrication.^5^ Dental gypsum waste disposal poses significant environmental challenges. Under anaerobic conditions in disposal sites, this mineral waste undergoes degradation, leading to the generation of toxic hydrogen sulfide gas and accelerating methane production. Therefore, finding alternative pathways for dental material recovery is essential to minimize these environmental risks.^4^ They can be recycled or reused in various fields following a calcination process with careful consideration of different calcination temperatures and holding times^6–8^ used a 20% w/v ammonium bicarbonate solution to completely dissolve the plaster of Paris (POP) residues. This process generates non-hazardous, useful by-products: ammonium sulfate for fertilizer and calcium carbonate for construction additives. Historically, the conversion of gypsum-based calcium sulfate dihydrate (DH) into functional bioceramics progressed *via* low-temperature dissolution–precipitation pathways. These methods primarily utilized ammonium or sodium phosphate routes to isolate highly crystalline β-tricalcium phosphate (β-TCP)^9,10^ and hydroxyapatite (HAp).^11^ Building on these low-temperature liquid techniques, researchers successfully transformed structured gypsum into advanced biomaterials. Lowmunkong *et al.*^12^ converted 3D-printed gypsum models into HAp by treating them directly in an ammonium phosphate solution. Barbosa *et al.*^13^ produced porous hydroxyapatite (HAp) prototypes from a 15% PVA–gypsum composite. This was achieved through chemical conversion in an (NH_4_)_2_HPO_4_ solution sustained at a pH of 6.0–9.0. Similarly, Thammarakcharoen *et al.*^14^ demonstrated that binder jet 3D-printed calcium sulfate bone grafts can be transformed into high-strength hydroxyapatite (HAp) simply by immersion in a phosphate solution. In a pioneering study, Han-Cheol *et al.*^15^ introduced an eco-friendly method to upcycle dental plaster waste into high-value β-tricalcium phosphate (β-TCP). Their approach transforms surplus calcium sulfate dihydrate (β-DH) by mixing it with zinc phosphate cement liquids and firing the compound above 900 °C to synthesize biocompatible bone substitutes. Due to a highly fragmented network of private laboratories, Tunisia lacks national monitoring for dental plaster models (DPMs). Furthermore, because these gypsum matrices pose no immediate toxic or infectious threats, regional management systems frequently neglect them in green dentistry initiatives.^16^ Instead, they are simply aggregated under broader healthcare or inert waste categories. This systemic oversight is reinforced by decree no. 2008-2745,^17^ which classifies clinical byproducts strictly by general hazard thresholds without allocating unique tracking codes to dental gypsum. Consequently, the identification and proper disposal of these materials remain deeply inadequate. To resolve this regulatory and environmental gap, this study introduces a sustainable valorization strategy to upcycle Tunisian dental plaster model waste (DPMW) into β-tricalcium phosphate (β-TCP). As a premier bioresorbable bioceramic that structurally mimics natural bone mineral, β-tricalcium phosphate plays a vital role in bone tissue engineering. Upon implantation, it actively drives hard-tissue regeneration by fostering osteoblastic cell proliferation and transitioning into a bioactive hydroxyapatite (HAp) layer upon exposure to physiological fluids, establishing an efficient, circular pathway for transforming localized dental waste into high-value biomedical implants.^18–20^ To maximize process efficiency, we employed a Box–Behnken design (BBD) integrated with response surface methodology. Since its discovery in 1960,^21,22^ Box–Behnken Design (BBD) has become a statistical cornerstone for quantitative optimization across chemical, environmental, and materials sciences.^23–28^ Unlike traditional downcycling methods like simple re-calcination,^4^ previous literature has demonstrated the feasibility of converting various forms of gypsum into calcium phosphates, these attempts have largely relied on empirical, single-variable approaches. To the best of our knowledge, this study is the first to implement a structured Design of Experiments framework, specifically a Box–Behnken Design (BBD) in order to evaluate and optimize the synthesis of β-tricalcium phosphate from dental plaster prosthesis waste. The novelty of this work resides not only in utilizing this specific, additive-rich dental waste matrix but in establishing a precise mathematical response surface model. This methodology is crucial for uncovering the complex synergetic effects of processing parameters, shifting the synthesis from empirical trial-and-error to a predictable and optimized recycling method for β-tricalcium phosphate. An in-depth comparative analysis was conducted on the processed material and raw gypsum waste to confirm this transformational process. Analysis included X-ray powder diffraction, Fourier-transform infrared spectroscopy, scanning electron microscopy with energy-dispersive X-ray spectroscopy, particle-size distribution, and dilatometric analysis.

## Experimental

### Materials and methods

The dental plaster model waste (DPMW) was kindly supplied by a dental prosthesis laboratory based in Sfax-Tunisia. Before use, the dental plaster model waste was dried in an oven at 80 °C for 24 h. After cooling, it was crushed in a brass mortar with a pestle, followed by grinding in a porcelain mortar for 15–30 minutes. The resulting powder was sieved with a stainless-steel sieve (*ϕ* < 150 µm) and labeled as DPMWP-150. Phosphoric acid with (H_3_PO_4_, purity >85%) was purchased from Sigma-Aldrich. Commercial β-tricalcium phosphate, used as a reference material, (labeled S_0_) was also purchased from Aldrich (purity > 98%).

### Synthetic procedure

**Scheme 1:**
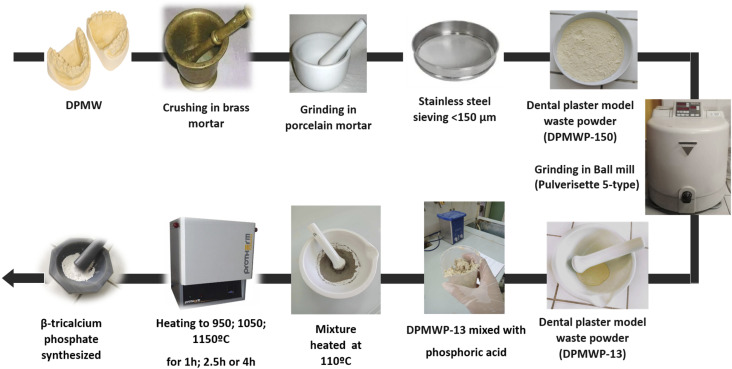
The different preparation stages of β-TCP from dental plaster model waste using phosphoric acid.

For the dry grinding of dental plaster model waste powder (DPMWP-150), a Fritsch laboratory planetary ball mill (Pulverisette 5, Fritsch, Spain) equipped with two stainless steel vessels and grinding balls (5 mm or 10 mm in diameter) was used. Regardless of the size of the balls, the total weight of the bowl and the balls was 2.36 kg. Each milling cycle processed about 36 g of dental plaster model waste powder (DPMWP-150). Standard atmospheric conditions were used for the grinding process, and the milling times were set at either 30 minutes at 100 rpm or 2 minutes at 360 rpm. Between successive grinding cycles, ventilation intervals of two to three minutes were included to prevent excessive heat buildup in the grinding bowls. Dental plaster model waste powder (DPMWP-13) was the name given to the powder produced after the milling process was optimized. Initially, for a standard batch, a stoichiometric volume of acid is combined with dried dental plaster model waste powder (DPMWP-13; *D*_50_ = 13.14 µm) in a set solid/liquid (S/L) ratio. The mixture was dried overnight at 110 °C and thereafter heated to the target temperature at a rate of 10 °C min^−1^. Scheme 1 describes the various preparation stages of β-tricalcium phosphate derived from dental plaster model waste powder. The theoretical reaction based on calcium sulfate precursor, intended to illustrate the detailed synthesis process, is given by eqn (1):

1 2H_3_PO_4_ + 3CaSO_4_·2H_2_O → 3H_2_SO_4_ + Ca_3_(PO_4_)_2_ + 6H_2_O

### Characterization

Phase composition and crystalline structures of dental plaster model waste powder (DPMWP-13), synthesized β-tricalcium phosphate (β-TCP), and commercial β-tricalcium phosphate were evaluated using a Bruker D8 ADVANCE X-ray powder diffractometer. The instrument operated with CuKα radiation (*λ* = 1.54 Å) at 40 kV and 40 mA. Diffractograms were scanned across a 2*θ* range of 10–70° with a step width of 0.01°. Qualitative and quantitative phases were identified using X'Pert HighScore software. Surface morphology and microstructure were evaluated using a Hitachi S-3400N scanning electron microscope (SEM) operated at an accelerating voltage of 15 kV. To determine elemental composition and distribution across both the dental plaster model waste powder (DPMWP-13) precursor and synthesized β-tricalcium phosphate solids, the instrument was coupled with energy-dispersive X-ray spectroscopy (EDX), enabling precise localized chemical quantification of the processed materials. Fourier-transform infrared (FTIR) spectra were collected at room temperature using a PerkinElmer Spectrum UATR Two spectrometer equipped with an Attenuated Total Reflectance (ATR) accessory. The samples were analyzed in air over a spectral range of 4000 to 400 cm^−1^ at a resolution of 4 cm^−1^ and 16 scans. Dilatometric analysis was performed using a SETARAM SETSYS Evolution instrument to evaluate the material's thermal behavior. Thermograms were dynamically recorded from room temperature up to 1300 °C under a constant heating rate of 10 °C min^−1^, ensuring accurate tracking of dimensional changes during the sintering process. The particle size distribution of dental plaster model waste powder (DPMWP) was analyzed using a Malvern Instruments Mastersizer 2000 Particle Size Analyzer (Model APA2000) with a Scirocco Dispersion Unit (Model ADA200). The standard volume percentiles *D*_10_, *D*_50_ and *D*_90_, were recorded and used to calculate the width of the distribution or ‘’span’’ as shown in eqn (2):2
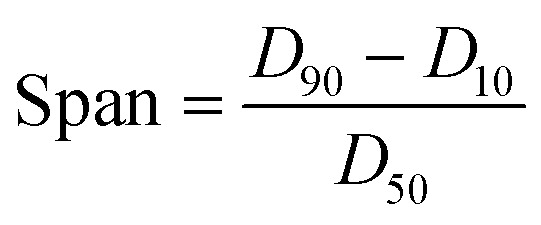


### Experimental design

**Fig. 1:**
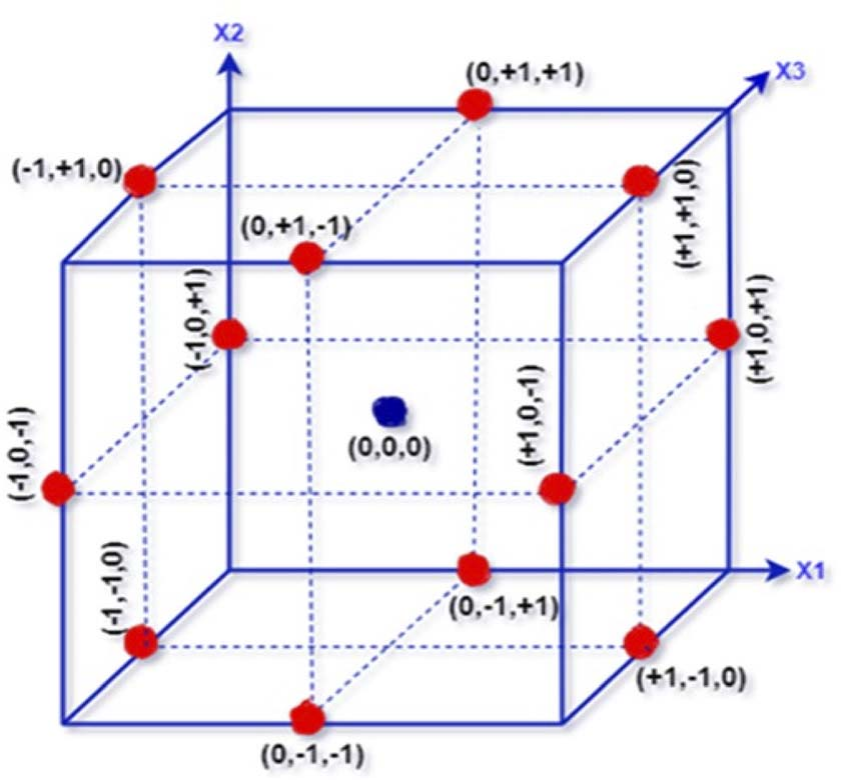
Geometric positions of experimental points for Box–Behnken design.

The Box–Behnken design optimizes and minimizes the number of experiments while accounting for possible interactions between the Box–Behnken design optimizes and minimizes the number of experiments while accounting for possible interactions between the factors under study and their effects on responses.^29,30^ Three key factors were selected for detailed study, designated as *X*_1_, *X*_2_ and *X*_3_. These parameters were varied across three levels: low (−1), medium (0) and high (+1), as shown in Table 1. The geometry of the Box–Behnken design with the selected factors is illustrated in Fig. 1.^30^ To estimate error, the central design point was repeated three times. An analysis of variance (ANOVA) was conducted to assess the model's validity and reliability.^31–33^ The NemrodW software was used for experimental design regression analysis and to derive the polynomial quadratic model.^34,35^ The Box–Behnken design (BBD) is a specific design within response surface methodology (RSM).^36,37^ Response surface methodology (RSM) optimization is a technique used to identify optimal values of independent variables that either maximize or minimize a response variable of interest. It combines statistical models, experimental design, and data analysis to establish a predictive relationship between the independent variables and the response variable.^38,39^

**Table 1:** Factors and their levels in Box–Behnken design

Code low	Factors	Low levels (−1)	Medium levels (0)	High levels (+1)
*X*_1_	S/L ratio	2.6	3.8	5
*X*_2_	Temperature (°C)	950	1050	1150
*X*_3_	Heating time (h)	1	2.5	4

The mathematical model commonly applied in RSM is a second-order polynomial equation, and its general form is given in eqn (3).3
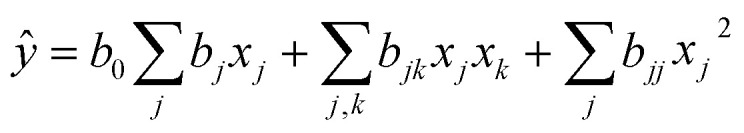
where *x*_*j*_ and *x*_*k*_ are the coded values of the independent factors, *ŷ* is the predicted response, *b*_0_ is the constant, and *b*_*j*_, *b*_*jk*_ and *b*_*jj*_ are the regression coefficients.^40^ The optimization *via* response surface analysis is considered successful, if the experimental and predicted results show strong agreement.

## Results and discussion

**Fig. 2:**
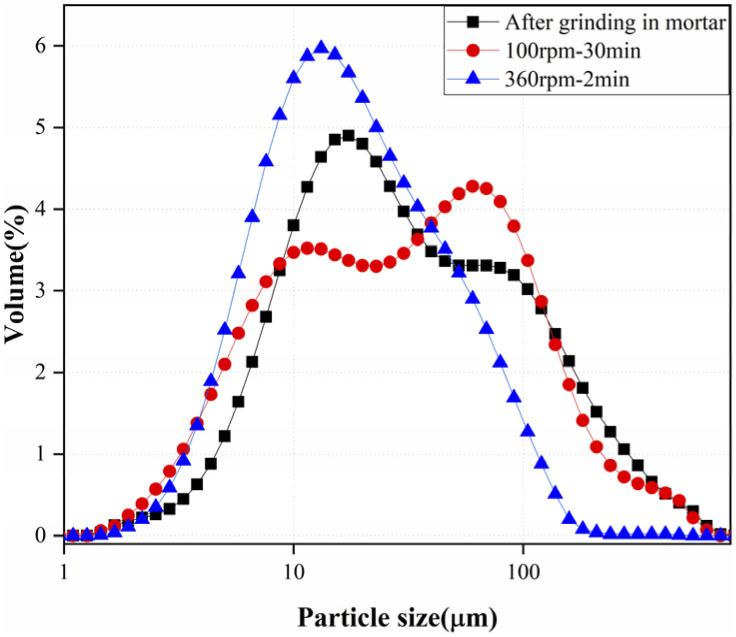
Particle size distribution of dental plaster model waste powder (DPMWP-150) after grinding in porcelain mortar and ball milling using two speeds.

The sample dental plaster model waste powder (DPMWP-150) underwent mechanical milling to establish a uniform particle size distribution, targeting a median diameter *D*_50_ of approximately 10 µm. High-energy planetary ball milling was initially performed utilizing large grinding media (*ϕ* = 10 mm) to evaluate the effects of rotational speed and duration. As illustrated by the granulometric profiles in Fig. 2, the unmilled dental plaster model waste powder (DPMWP-150) sample exhibited a distinct bimodal distribution consisting of two discrete particle populations: a fine fraction spanning 1–70 µm and a coarser agglomerate fraction ranging from 20 to 600 µm. Following low-speed milling at 100 rpm for 30 min, the distribution retained its bimodal character, though the boundary limits shifted to 1 to 23 µm and 11 to 700 µm for the primary and secondary populations, respectively. Conversely, escalating the milling intensity to 360 rpm for an abbreviated duration of 2 min effectively disrupted the structural agglomerates. This high-speed configuration eliminated the bimodal distribution, yielding a monomodal particle profile with a significantly reduced *D*_50_ of 18.16 µm. In the second stage, we set the milling time to 2 minutes and the speed to 360 rpm, varying the size of the balls (big balls *ϕ* = 10 mm, small balls *ϕ* = 5 mm and a mixture of both sizes).

**Fig. 3:**
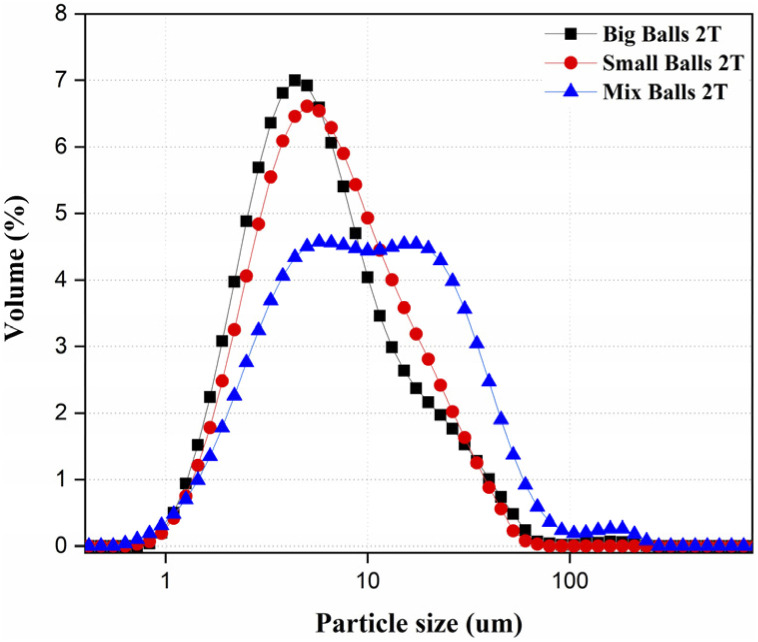
Particle size distribution of dental plaster model waste powder (DPMWP-150) after ball milling for 2 min at speed 360 rpm using 2 turns and different ball sizes.

**Fig. 4:**
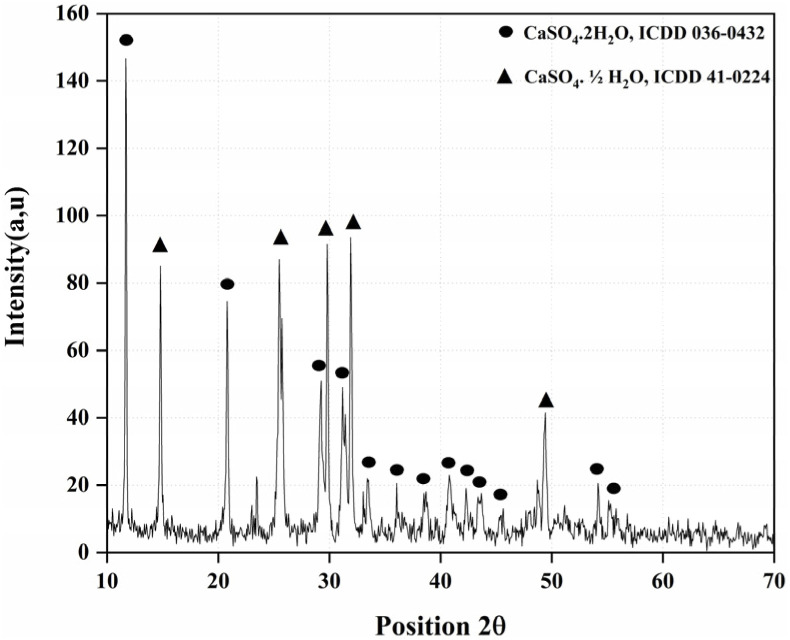
XRD pattern of the starting material dental plaster model waste powder (DPMWP-13).

The grinding process was performed once and twice under the same conditions. The results of this study are shown in Table 2 and Fig. 3. The finest powder of PSD results was achieved using big balls and performing two cycles at 360 rpm, resulting in an almost single population distribution ranging from 0.1 to 30 µm, with a *D*_50_ value of 13 µm. Fig. 4 displays the XRD pattern of the dental plaster model waste powder (DPMWP-13) precursor (*D*_50_ = 13 µm) where sharp Bragg reflections signify highly crystalline phases. Phase quantification *via* HighScore software reveals a biphasic composition consisting of 58% calcium sulfate dihydrate (DH; CaSO_4_·2H_2_O, ICDD 33-0311) and 42% calcium sulfate hemihydrate (HH; CaSO_4_·½H_2_O, ICDD 41-0224).^40,41^ The diffraction peaks for calcium sulfate dihydrate (DH) emerge at 11.64°, 20.75°, 23.41°, and 29.14°, corresponding to the (020), (021), (040), and (041) planes, respectively. Concurrently, distinct peaks at 14.76° (110), 25.67° (310), 29.77° (220), and 31.91° (−114) index to calcium sulfate hemihydrate (HH). Ultimately, the persistence of unreacted calcium sulfate hemihydrate (HH) within the waste powder confirms an incomplete hydration setting mechanism. This process is expressed by eqn (4):

4 CaSO_4_·½H_2_O + 3/2H_2_O → CaSO_4_·2H_2_O

**Table 2:** Experimental parameters for ball milling DPMWP-150

Cycles number	1 Cycle				2 Cycles			
Ball size	*D*_10_	*D*_50_	*D*_90_	Span	*D*_10_	*D*_50_	*D*_90_	Span
Big (*d* = 10 mm)	6.30	18.16	67.19	3.35	5.30	13.14	50.53	3.44
Small (*d* = 5 mm)	6.22	18.56	61.25	2.96	5.72	15.03	50.10	2.95
(1/2 big + 1/2 small)	6.10	20.50	79.51	3.59	6.31	23.69	88.03	3.45

**Fig. 5:**
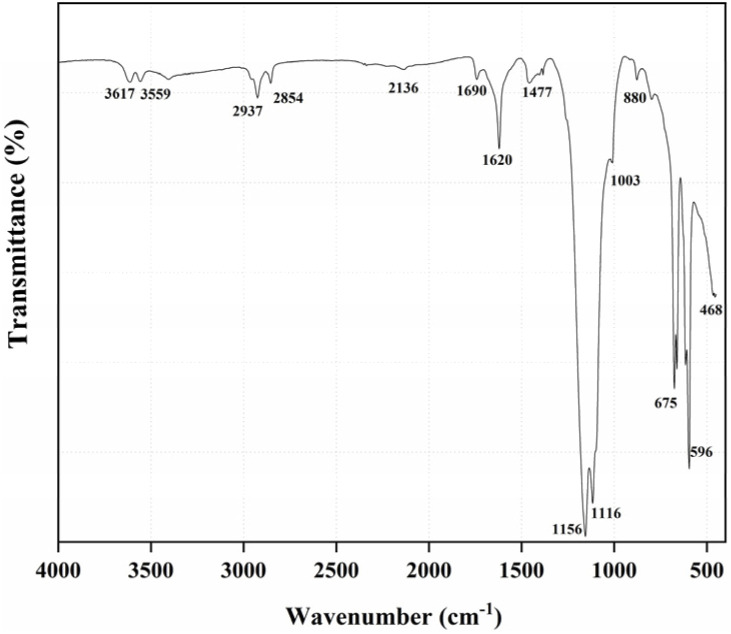
The FTIR spectra of dental plaster model waste powder (DPMWP-13).

**Fig. 6:**
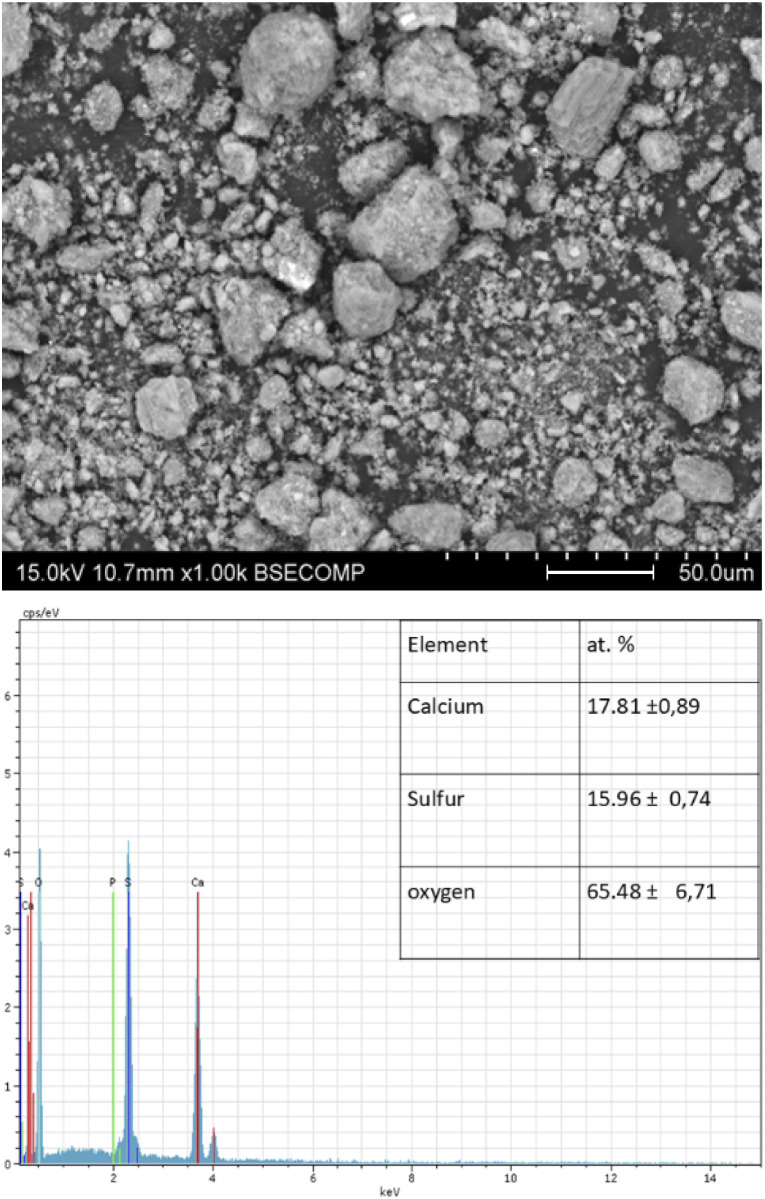
SEM micrograph and EDX spectrum of dental plaster model waste powder (DPMWP-13).

Fig. 5 shows the FTIR spectra of dental plaster model waste powder (DPMWP-13). According to the literature, the bands identified in the FTIR spectrum are typical of calcium sulfate.^42^ We observe two main regions: one related to O–H bond vibrations in water molecules and another to the sulfate vibration bands. Additionally, carbonates in the sample are indicated by bands at 1477 cm^−1^ and 880 cm^−1^. The region associated with the valence vibrations of the O–H bond in water is located around 3700–3300 cm^−1^. The bands at approximately 3617 cm^−1^ and 3559 cm^−1^ correspond to the stretching hydroxyl vibrations (*ν*_O–H_) of calcium sulfate hemihydrate (HH), while the absorptions at ∼3559 cm^−1^, ∼3404 cm^−1^, and ∼3240 cm^−1^ are attributed to *ν*_O–H_ in calcium sulfate dihydrate (DH). The bands at 1620 and 1690 cm^−1^ are linked to the deformation mode of O–H (*δ*_O–H_) in water molecules. The band around 1620 cm^−1^ indicates strongly held water molecules, whereas the 1690 cm^−1^ band (*δ*_O–H_) indicates the presence of loosely held water molecules in calcium sulfate dihydrate (DH) and is absent in calcium sulfate hemihydrate (HH). The Bands at 2854 and 2837 cm^−1^ indicate organic additives in the dental plaster,^42^ representing asymmetric and symmetric CH_2_ stretching.The 1450–1370 cm^−1^ region confirms these hydrocarbons through characteristic CH_2_ and CH_3_ bending and scissoring vibrations that typically accompany primary C–H stretches.^43^ The characteristic bands assigned to the sulfate group (SO_4_^2−^) vibrations appear in the 1200–450 cm^−1^ region.^35^ The vibrations of S–O bonds in SO_4_^2−^ are identified at 468, 696, 664, 1003, 1116, 1156 and 2236 cm^−1^.^43^ The small band around 468 cm^−1^ is attributed to *ν*_2_ (SO_4_^2−^) bending vibrations. The coexistence of calcium sulfate dihydrate (DH) and calcium sulfate hemihydrate (HH) causes a distinct splitting of the *ν*_4_ (SO_4_^2−^) bending mode into a doublet at 596 and 675 cm^−1^. The primary *ν*_3_ (SO_4_^2−^) stretching bands emerge at 1116 and 1156 cm^−1^. A minor *v*_1_ (SO_4_^2−^) stretching peak is resolved at 1003 cm^−1^. The band about 2236 cm^−1^ is attributed to the combination and overtones of SO_4_.^42^Fig. 6 displays the SEM micrograph alongside the corresponding EDX spectrum of dental plaster model waste powder (DPMWP-13). The microscopic image reveals pronounced particle agglomeration, characterized by heterogeneous clusters of small and large particles that consistently remain below 20 µm. Furthermore, the EDX elemental analysis confirms the co-existence of calcium, sulfur, and oxygen. The calculated Ca/S molar ratio of 1.12 closely aligns with the theoretical stoichiometric ratio of 1, thereby verifying the predominant presence of calcium sulfate within the waste powder matrix. Table 3 presents the fifteen experiments (*N* = 15), which include 12 design test points and three central points to estimate the experimental error. *X*_1_ represents the S/L ratio, *X*_2_ the temperature (°C), and *X*_3_ the heating time in hours. The response (*y*_1_) presents the Relative Crystallinity index (RCI,%) which is the area of the peak at 31.14° 2*θ* for each sample diffractogram, divided by area of the same peak in commercial β-TCP, as presented in the following equation eqn (5). The β-TCP peak at 31.14° is the most intense for this material.5
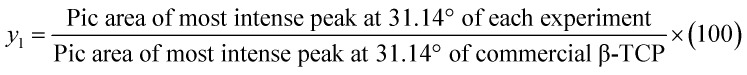


**Table 3:** The results of the Box–Behnken design experiments

Exp no.	*X*_1_	*X*_2_	*X*_3_	*y*_1_
S_1_	2.6	950	2.5	70.10
S_2_	5.0	950	2.5	64.95
S_3_	2.6	1150	2.5	81.02
S_4_	5.0	1150	2.5	86.62
S_5_	2.6	1050	1.0	87.42
S_6_	5.0	1050	1.0	53.30
S_7_	2.6	1050	4.0	59.12
S_8_	5.0	1050	4.0	41.64
S_9_	3.8	950	1.0	78.18
S_10_	3.8	1150	1.0	135.40
S_11_	3.8	950	4.0	83.37
S_12_	3.8	1150	4.0	102.43
S_13_	3.8	1050	2.5	131.18
S_14_	3.8	1050	2.5	123.37
S_15_	3.8	1050	2.5	131.97

A second order polynomial equation for (*y*_1_) with its coefficients was calculated using the least squares method:

6 *ŷ* = 128.840 − 6.394*X*_1_ + 13.609*X*_2_ − 8.467*X*_3_ − 46.321*X*_12_ − 6.846*X*_22_ − 22.149*X*_32_ + 2.688*X*_1_*X*_2_ + 4.160*X*_1_*X*_3_ − 9.540*X*_2_*X*_3_

ANOVA is a statistical technique that subdivides the total variation in a dataset into component parts associated with specific sources of variation, allowing for hypothesis testing on the model parameters.^34^

The statistical significance of the ratio of the mean square variation due to regression and the mean square residual error was tested using analysis of variance (ANOVA) as presented in Table 4 in relation to (*y*_1_). The total sum of squares (SS_tot_), with 14 degrees of freedom (dof), is divided into two sums of variation: SS_Reg_ due to regression with 9 degrees of freedom, and SS_Res_, related to residuals with 5 degrees of freedom. Table 3 The results of the Box–Behnken design experiments.

7 SS_Tot_ = SS_Reg_ + SS_Res_

**Table 4:** Analysis of variance (ANOVA) of the Box–Behnken design

Source of variation	SS	Dof	MS	*F*-Value	Significance
Regression	12 064.1	9	1340.46	10.454	0.937**
Residual	641.091	5	128.219		
Validity	595.541	3	198.64	8.897	10.4
Error	45.15	2	22.57		
Total	12 705.2	14			

The adequacy of the model is evaluated by comparing the regression variance with the residual variance.8
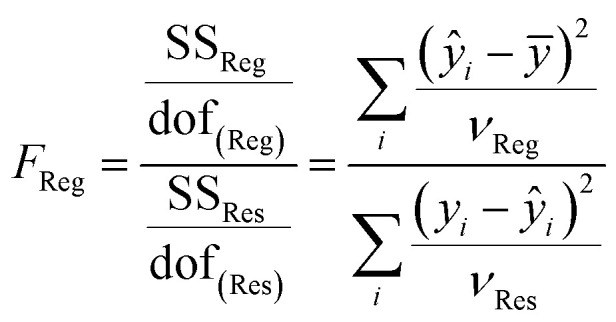


According to Table 4, *F*_Reg_ = 10.45 exceeds the Fisher critical value for a 99% confidence level (*F*_0.01_ (9.5) = 10.16), indicating that the regression is significant. The multiple correlation coefficient *R*^2^ = 0.950 confirms the adequacy of the model. The residual sum of squares is further divided into the variation due to pure experimental error (SS_Error_) with 2 degrees of freedom (using the central experiments) and the “lack of fit” (SS_lof_) with 3 degrees of freedom, which is used to evaluate the validity of the model.

9 SS_res_ = SS_E_ + SS_lof_

**Fig. 7:**
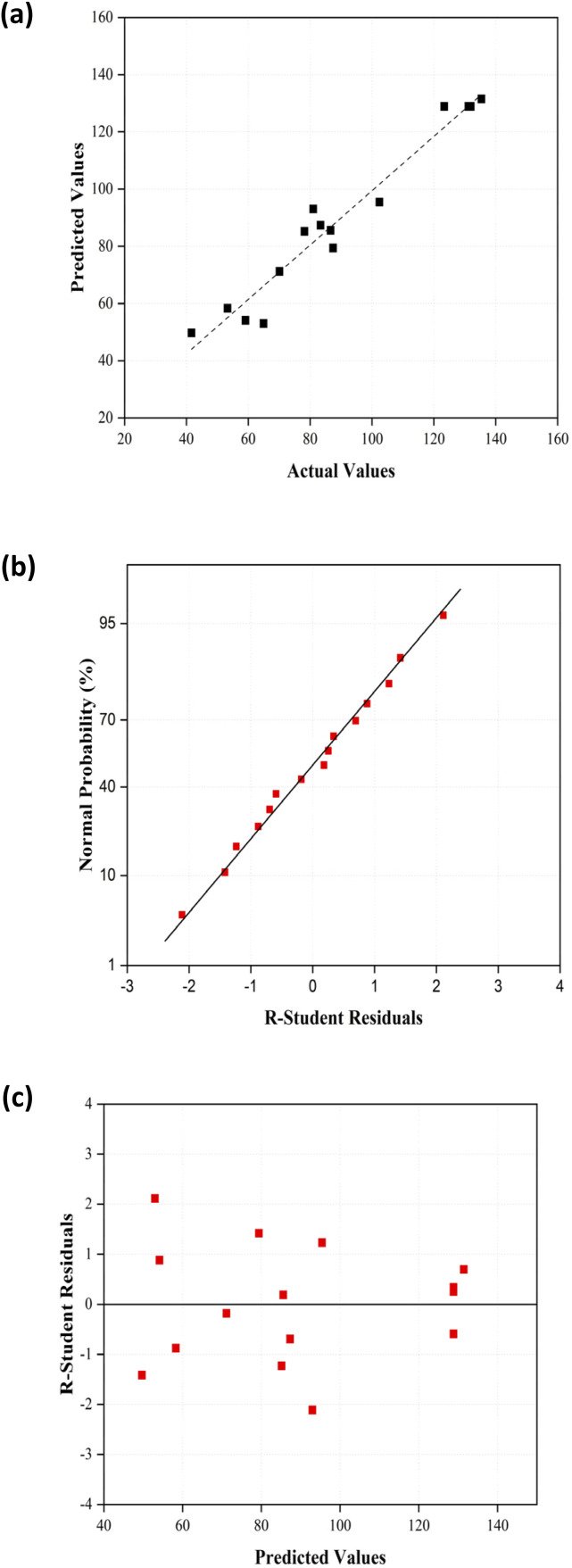
Diagnostic plots of model adequacy: (a) predicted *versus* actual plots, (b) normal probability plots of residuals, and (c) *R*-Student residuals *versus* predicted values.

For model validation, the error variance is compared with the variance due to lack of fit.10
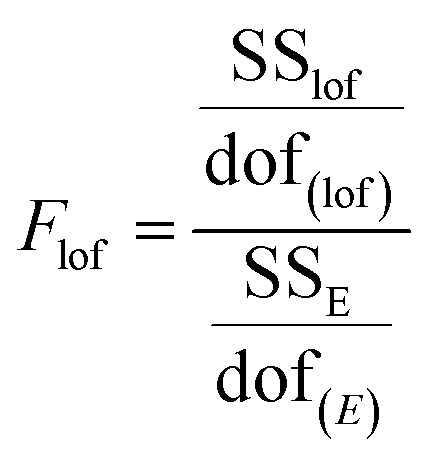
*F*_lof_ = 10.4 is below Fisher's critical value for a 95% confidence level (*F*_0.05_ (3.2) = 19.16). This indicates that the lack of fit is of the same order of magnitude as the pure error, confirming that the model is valid. In conclusion, the proposed model is adequate and valid for describing the response. It can be used as a predictive equation within the studied domain. In order to evaluate and predict the individual and interactive effects of the three independent processing variables—solid-to-liquid ratio, sintering temperature, and heating time as well as to optimize the crystalline response of the synthesized β-tricalcium phosphate, all experimental trials were furthermore, the model's predictive capability and reliability were verified by a predicted *R*-squared-pred *R*^2^-squared of 0.242, a PRESS statistic of 9636.660 and a standard of deviation of 11.323 over 5 degrees of freedom as indicated in Table 5. Fig. 7 displays the statistical validation plots for the developed BBD model. Fig. 7(a) depicts the predicted *versus* actual values of the response *y*_1_. As shown, the data points are closely distributed along the straight diagonal line, proving an excellent agreement between the experimental and predicted values, which confirms the reliability of the model. Furthermore, the normal probability plot of the residuals depicted in Fig. 7(b) indicates a straight-line trend. This implies that the underlying errors follow a normal distribution with no severe outliers, thereby satisfying the fundamental assumption of the analysis of variance (ANOVA). Deviations into an S-shape are absent, confirming that no mathematical transformation of the response variable is required. Finally, Fig. 7(c) displays the *R*-Student residuals *versus* the predicted *y*. The data points exhibit a completely random distribution above and below the zero-horizontal line, with all residuals falling well within the acceptable limits of ±3. This random scattering demonstrates that the variance of the errors is constant and confirms that the proposed model accurately depicts the optimization procedure without any systematic errors. The regression coefficients, along with their corresponding *t*-values, *p*-values, and 95% confidence intervals (CI), are summarized in Table 6. The significance of each coefficient was evaluated using its *p*-value at a 95% confidence level. Statistically, a factor is considered highly significant if its *p*-value is less than 0.05, and its 95% CI does not include zero.

**Table 5:** Model fit summary and validation statistical parameters

Standard deviation of the response	11.323
*R* ^2^-squared	0.950
Adj *R*^2^-squared	0.859
Pred *R*^2^-squared	0.242
PRESS	9636.660
Degrees of freedom	5

**Fig. 8:**
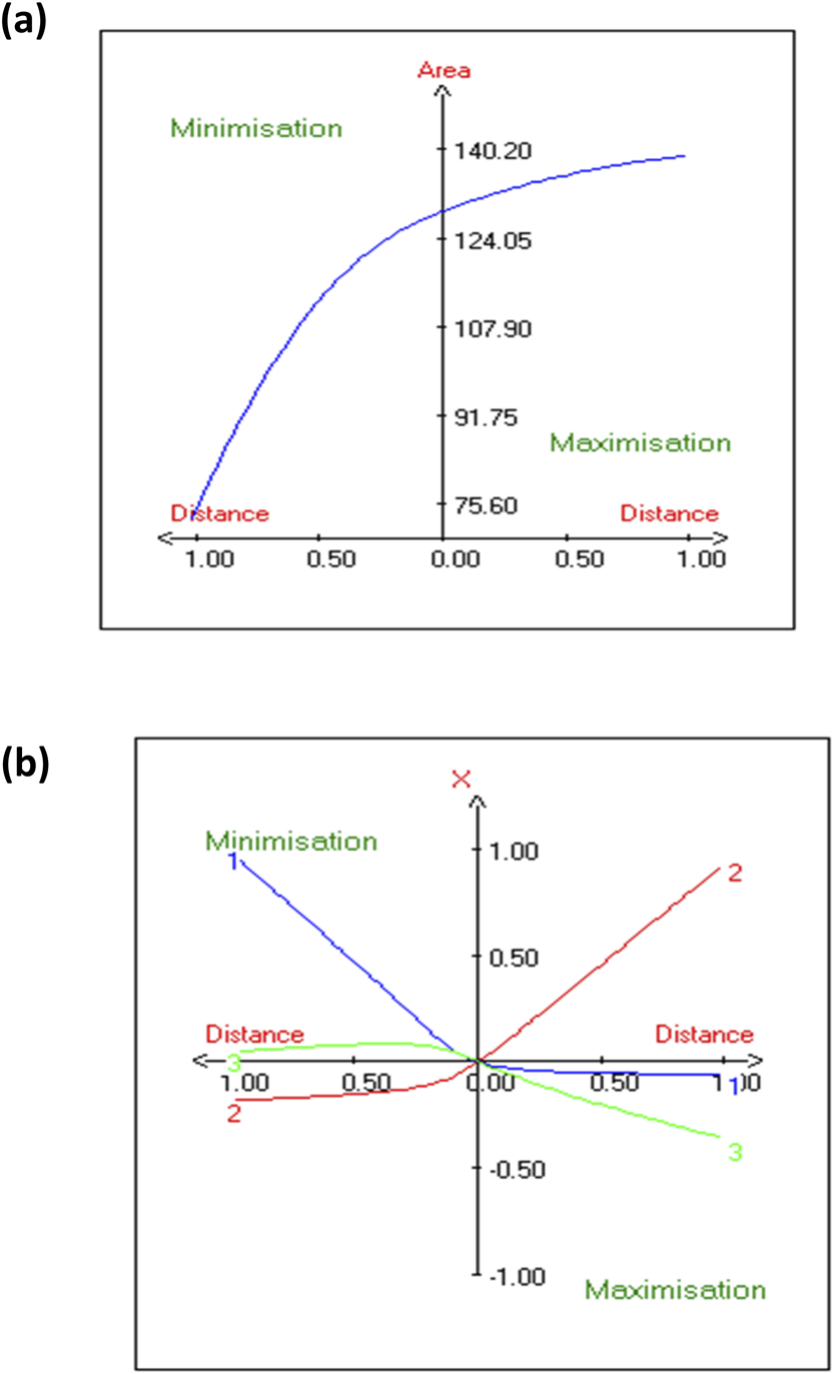
Optimal path for the response *y*_1_: (a) the maximum response value, (b) the corresponding coded variables.

**Fig. 9:**
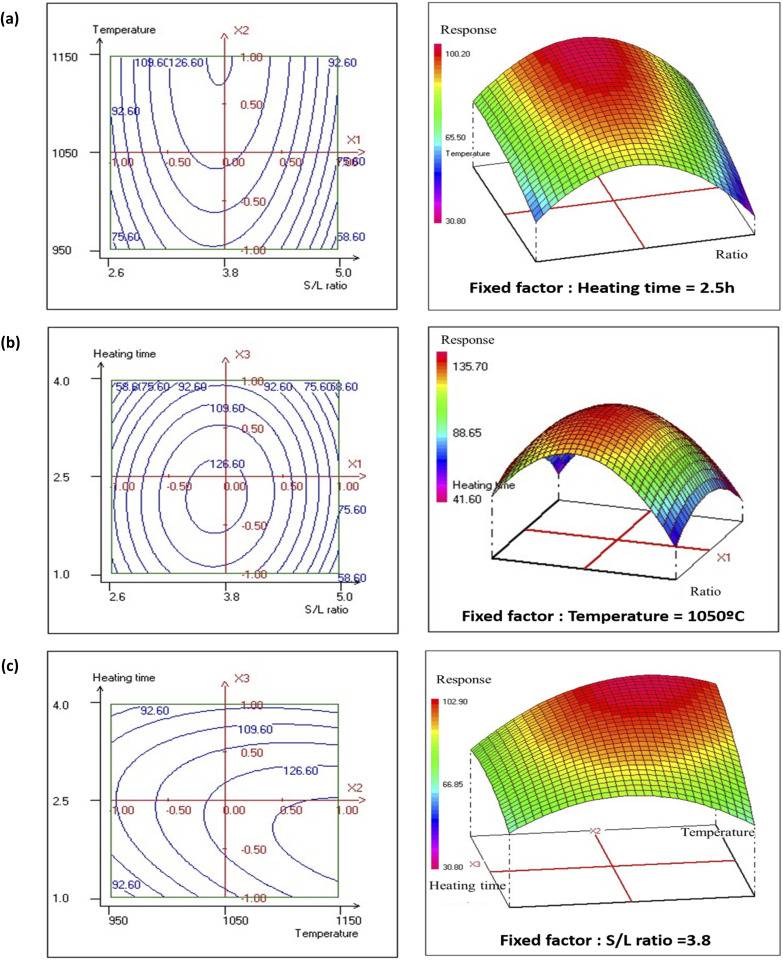
Two-dimensional contour and three-dimensional surface plots depicting the factor interactions on the β-TCP peak area response: (a) interaction between S/L ratio and temperature, while keeping heating time constant at 2.5 h; (b) interaction between heating time and S/L ratio, at a fixed temperature of 1050 °C; and (c) interaction between temperature and heating time, with the S/L ratio fixed at an optimal value of 3.8.

**Fig. 10:**
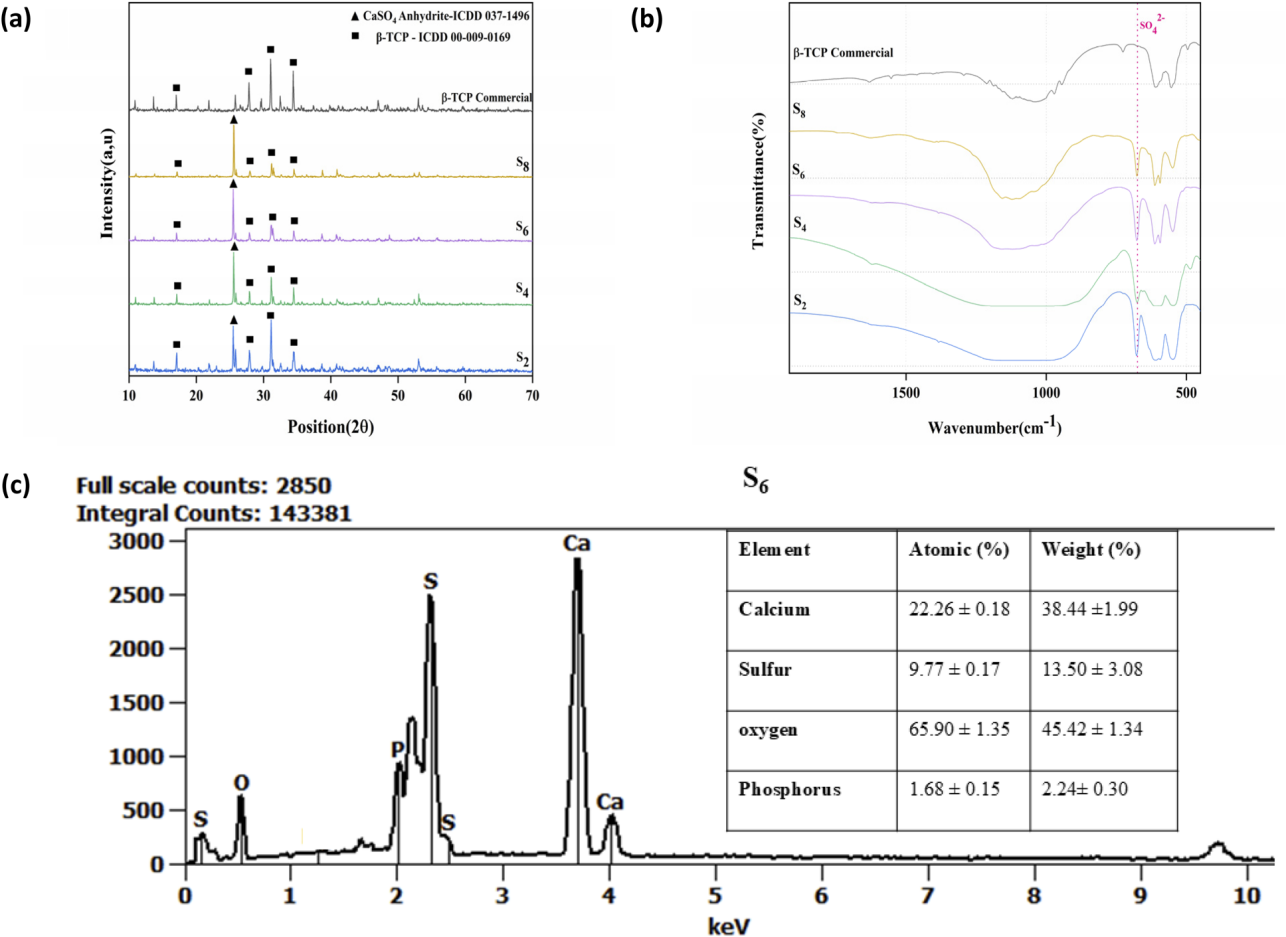
(a) XRD profiles (b) FTIR spectra of S_2_, S_4_, S_6_ and S_8_ solids using S/L = 5 and different sintering temperatures and dwell times (c) EDX analysis of S_6_ solid.

**Fig. 11:**
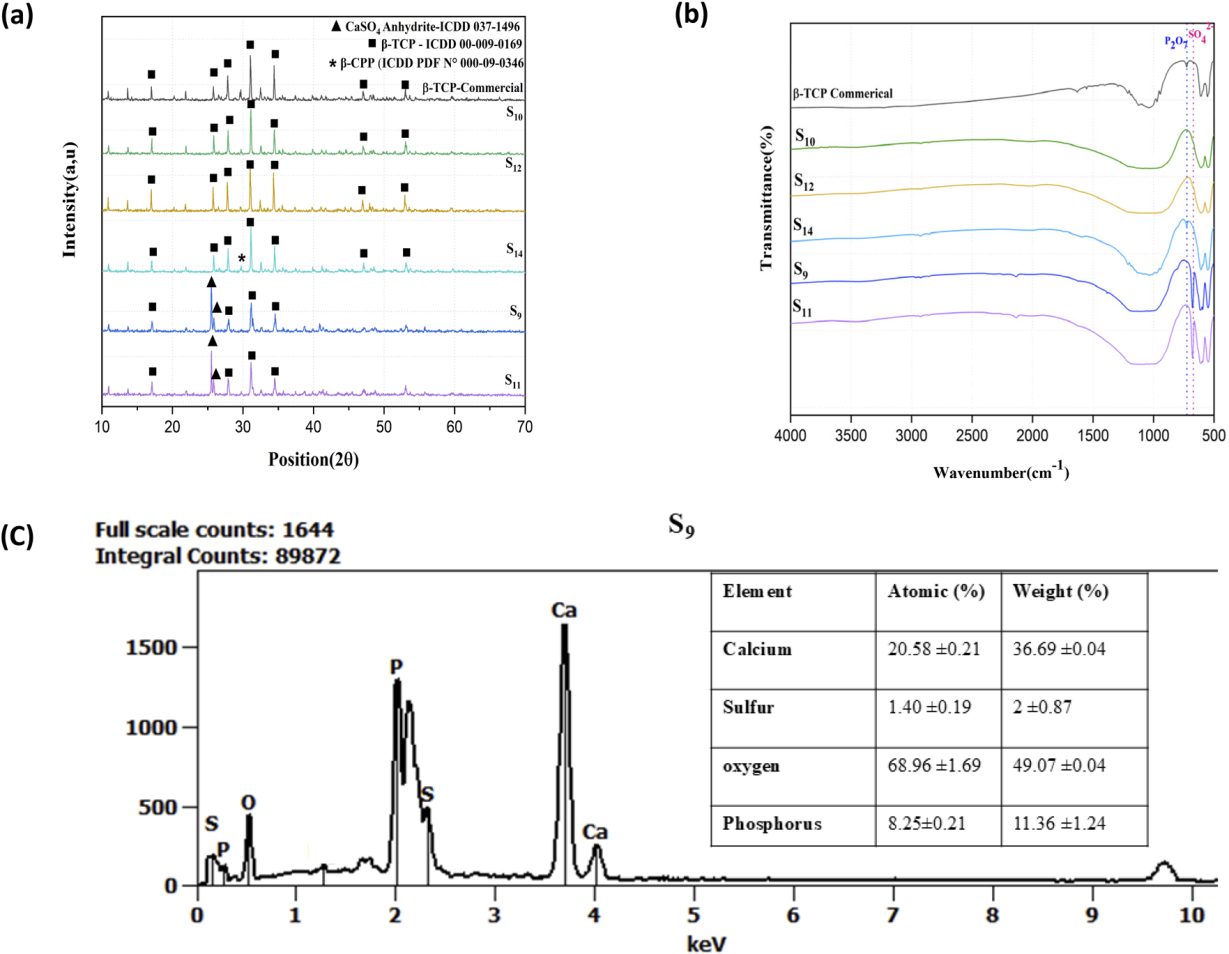
(a) XRD profiles and (b) FTIR spectra of S_9_, S_10_, S_11_, S_12_ and S_14_ solids using S/L = 3.8 and different sintering temperatures and dwell times and (c) EDX analysis of S_9_.

**Fig. 12:**
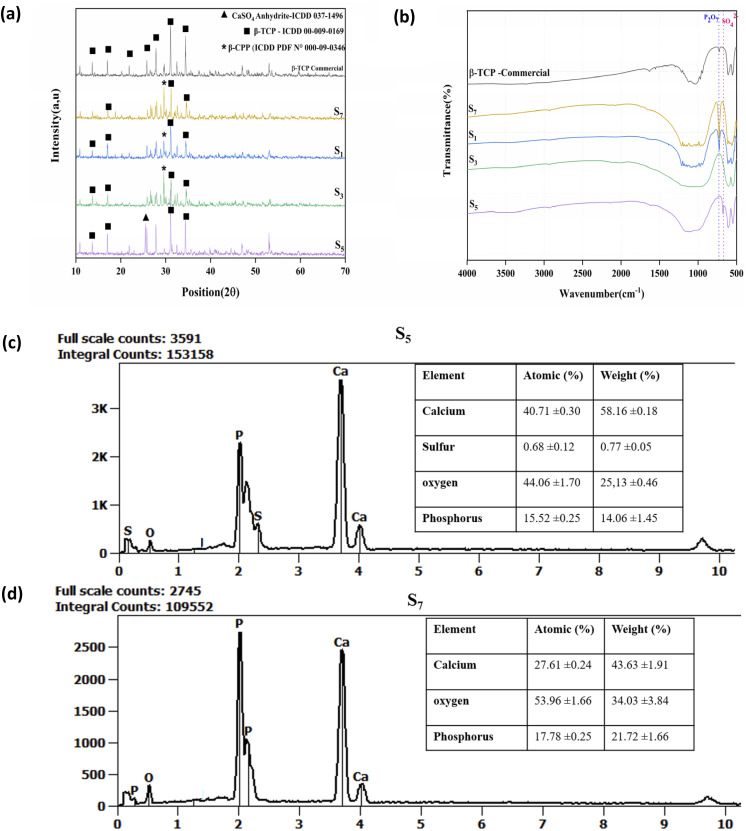
(a) XRD profiles (b) FTIR spectra of S_1_, S_3_, S_5_ and S_7_ solids using S/L = 2.6 and different sintering temperatures and dwell times (c) EDX analysis of S5 (d) EDX analysis S_7_.

Based on these criteria, the intercept (*b*_0_), as well as the terms corresponding to *b*_2_ and *b*_33_, demonstrated significant effects on the response variable (*p* < 0.05). Conversely, the remaining terms exhibited *p*-values greater than 0.05 and confidence intervals spanning across zero, indicating that their effects are statistically non-significant within the investigated experimental domain. The model's exploitation is conducted through the analysis of the optimal path, isoresponse curves, and response surfaces. As illustrated in Fig. 8, analyzing the optimal path allows for the determination of the precise coordinates where the desired response is maximized on any hypersphere of radius *R* centered at the origin. The trajectory of these successive points constitutes the optimal path itself. This path serves a dual purpose: it tracks the shifting optimal values of the response at expanding distances from the center while simultaneously identifying the corresponding optimal levels for each experimental factor. According to Fig. 8(a), the maximum response value (139%) corresponds to a distance of 1.0 from the origin. Fig. 8(b)) indicates the corresponding coded variables (*X*_1_ = −0.1, *X*_2_ = +1 and *X*_3_ = −0.3 which translate to the following experimental parameters: S/L ratio of 3.7, a temperature of 1150 °C and a heating time of 2.2 h. Among the experimental runs, S_10_ is the closest to these predicted optimum conditions and exhibits a relative crystallinity index higher than that of the commercial reference material. Therefore, it was selected as the optimum experimental condition investigated in this study. Fig. 9 summarizes the results and the impacts of interactions using 2D contour plots and 3D surface plots. As demonstrated in Fig. 9(a), at a fixed heating time of 2.5 hours, elevating the sintering temperature promotes an increase in the characteristic peak area at 31.14° 2*θ*. Concurrently, the response rises alongside the S/L ratio, reaching a distinct maximum at an optimal ratio of 3.8 before undergoing a subsequent decline. This parabolic behavior is corroborated by Fig. 9(b), which indicates that at a constant temperature of 1050 °C, the response peaks at critical thresholds of a ratio S/L = 3.7 and 2.2 hours of thermal treatment. Similarly, Fig. 9(c) reveals that while higher temperatures consistently enhance the peak area, the prolonged heating time only benefits the crystalline response up to 2.2 hours, after which a gradual decrease occurs. Fig. 10–12 show the XRD profiles, FTIR spectra and EDX analyses of the prepared samples prepared samples using different S/L ratios, sintering temperatures and dwell times. According to Table 3, it is noted that at 950 °C the anhydrite CaSO_4_ is present in all experiments whatever the S/L ratio and the dwell time. The anhydrite CaSO_4_ derives from the total dehydration of the CSD at 950 °C.

**Table 6:** Regression coefficients, *t*-values, *p*-values, and 95% confidence intervals of the predicted model

Parameter	Coefficient	Standard error	*t*-Value	*p*-Values	Confidence interval
*b*_0_	128.846	6.538	19.71	0.01	[115.770; 141.922]
*b*_1_	−6.395	4.003	−1.60	0.171	[−14.401; 1.611]
*b*_2_	13.607	4.003	3.40	0.193	[5.601; 21.613]
*b*_3_	−8.468	4.003	−2.12	0.088	[−16.474; −0.462]
*b*_11_	−46.322	5.893	−7.86	0.01	[−58.108; −34.536]
*b*_22_	−6.848	5.893	−1.16	0.298	[−18.634; 4.938]
*b*_33_	−22.150	5.893	−3.76	0.132	[−33.936; −10.364]
*b*_12_	2.689	5.662	0.47	0.655	[−8.635; 14.013]
*b*_13_	4.164	5.662	0.74	0.495	[−7.160; 15.488]
*b*_23_	−9.537	5.662	−1.68	0.153	[−20.861; 1.787]

Using experiment S_9_ as an example, the EDX analysis shows that CaSO_4_ is present (*S* = 2% wt) as depicted in Fig. 11(C). In addition, in all experiments presented in Fig. 10, when S/L ratio is equal to 5, the anhydrite CaSO_4_ is also present whatever the reaction temperature. Semi-quantitative phase analysis using HighScore software revealed that the CaSO_4_ content remained consistently less than 60% in all experimental runs. Taking the example of experiment S_6_, which contains 13.5% wt of sulphur, as shown in Fig. 10(C) of the EDX analysis. This can be explained by the excess of CaSO_4_·2H_2_O compared to H_3_PO_4_ as shown by FTIR and XRD techniques. Nevertheless, with stoichiometric quantities of CaSO_4_·2H_2_O and H_3_PO_4_ (S/L = 3.8) and a temperature of 1050 °C, we obtain β-TCP contaminated with small quantity of calcium pyrophosphate β-CPP (β-Ca_2_P_2_O_7_). Semi-quantitative phase analysis using HighScore software revealed that the pyrophosphate content remained consistently below 9% across all these experimental runs. It is well known that Ca_2_P_2_O_7_ has three polymorphs: γ-Ca_2_P_2_O_7_, β-Ca_2_P_2_O_7_ and α-Ca_2_P_2_O_7_ which begin to crystallize from 270 to 430 °C, 750–850 °C and 1140–1220 °C, respectively.^37^

**Fig. 13:**
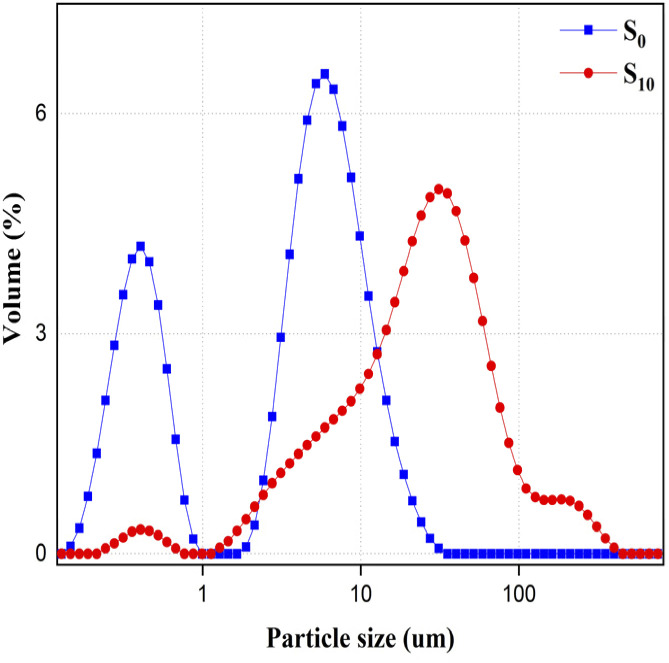
Particle size distribution of commercial β-TCP S_0_ and sample S_10_.

**Fig. 14:**
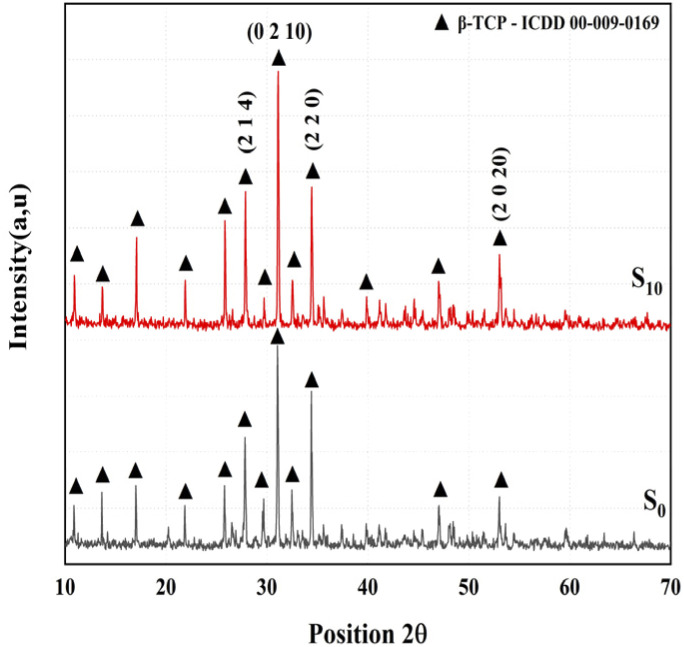
XRD analysis of commercial β-TCP (S_0_) and sample S_10_.

**Fig. 15:**
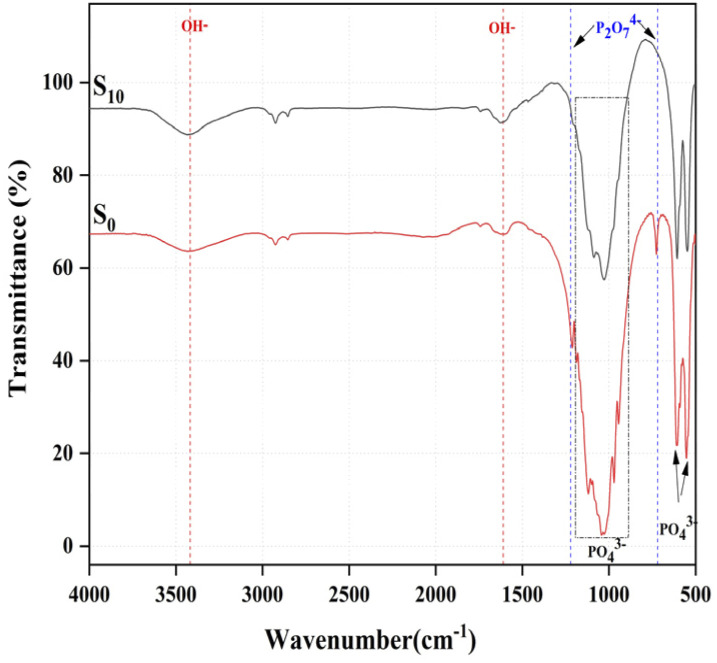
The FTIR spectra of commercial β-TCP S_0_ and sample S_10_.

**Fig. 16:**
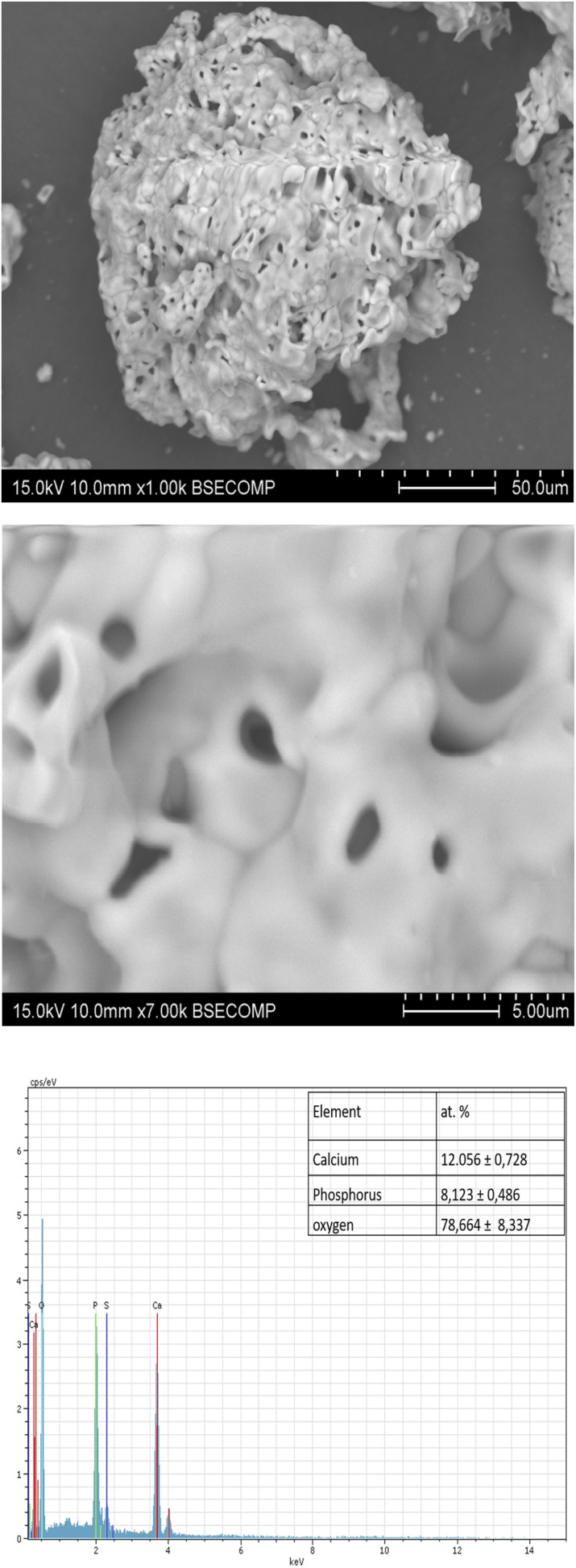
SEM micrographs (at 1000× and 7000× magnification) and corresponding EDX spectrum of the synthesized sample S_10_.

**Fig. 17:**
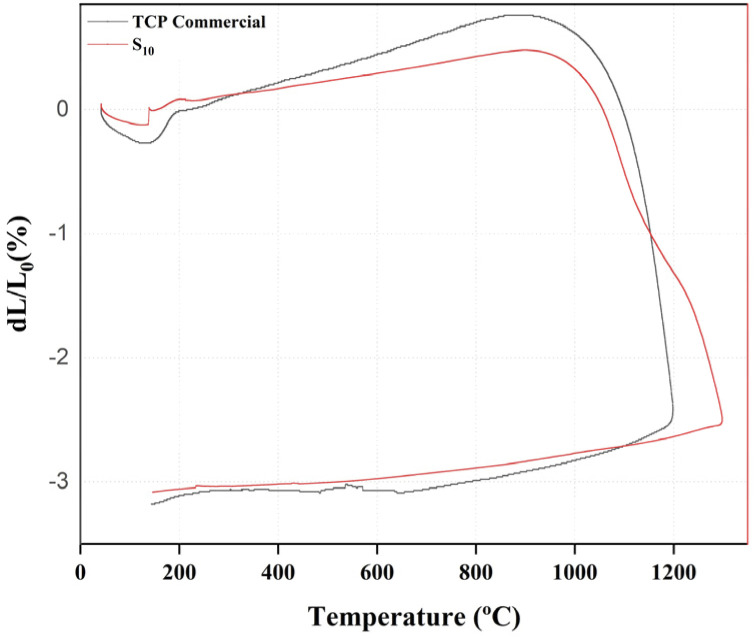
Dilatometric curve of commercial β-TCP S_0_ and sample S_10_.

On the other hand, when we increase the quantity of H_3_PO_4_ (S/L = 2.6) using different temperatures 950, 1050 and 1150 °C for 2.5 h and 4 h, we find β-TCP contaminated with pyrophosphate (β-CCP). Semi-quantitative phase determination using HighScore software revealed that the pyrophosphate percentage is verified to be less than 54%. However, the EDX spectra in Fig. 12(C), representing experiments S_5_ and S_7_, reveal that CaSO_4_ is present in experiment S_5_ but absent in experiment S_7_. This observation can be explained by the difference in sintering time, experiment S_5_ having been sintered for 1 hour, whereas experiment S_7_ benefited from a longer sintering time of 4 hours. Based on the Box–Behnken design (Table 3). As known, S_10_ was selected as our practical optimum, as it empirically provides the best operating conditions to successfully synthesize β-TCP. This sample underwent comprehensive characterization *via* XRD, FTIR, SEM, EDX, and dilatometric analysis. To evaluate its quality, the results of S_10_ were compared to S_0_, a commercial β-TCP. As shown in Fig. 13, both of commercial β-TCP (S_0_) and the experimental sample S_10_ displayed bimodal particle size distributions. The samples featured two distinct populations. The first population was identical for both S_0_ and S_10_. It spanned a range of 0–1 µm with a mode (M_1_) of 0.5 µm. For S_0_, the second population ranged from 1.5 to 40 µm with a mode (M_2_) of 6 µm. In contrast, S_10_ exhibited a much broader range of 1–170 µm and a higher mode of 31 µm. Fig. 14 shows the XRD patterns of S_0_ and experiment S_10_. Sample S_10_ was prepared with an S/L ratio of 3.8 and fired at 1150 °C for 1 hour. The profile of S_10_ perfectly matches the standard XRD pattern of β-TCP (ICDD PDF no. 00-009-0169).^38^ Furthermore, its crystallinity exceeds that of commercial β-TCP. The product yield is 99.888%, with 12 g of β-TCP obtained from 20 g of dental plaster model waste. This conversion yield is consistent with the X-ray diffraction (XRD) data, as there are no traces of dental plaster model. Fig. 15 displays the FTIR spectra for samples S_0_ and S_10_. In both spectra, the distinct bands at 550, 600, 925, 975, 1050, and 1125 cm^−1^ confirm the presence of PO_4_^3−^ groups in β-TCP. Additionally, absorption bands at 725, 1200, and 1225 cm^−1^ signify calcium pyrophosphate (β-Ca_3_(PO_4_)_2_) impurities, whereas the peaks at 3450 and 1640 cm^−1^ correspond to adsorbed water.^44^ Notably, sample S_10_ exhibits a lower concentration of calcium pyrophosphate phases compared to the commercial β-TCP (S_0_). These spectral profiles align well with previously published literature.^45–47^ The SEM micrograph and EDX analysis of sample S_10_ are presented in Fig. 16. The micrograph reveals fused blocks. These blocks exhibit a distinct coral-like structure after firing at 1150 °C. The EDX analysis identified several key elements. It confirmed the presence of calcium, phosphorus, sulfur, and, and oxygen. The calculated molar Ca/P ratio is 1.48. This result is very close to the stoichiometric value for β-TCP, which is 1.50. A small quantity of sulfur was also detected. This presence can be attributed to trace amounts of anhydrite CaSO_4_. Alternatively, it may result from insufficient washing of the sample. This step is required to remove H_2_SO_4_ formed during the reaction according to eqn (1). Notably, our SEM micrographs exhibit close similarities to those reported in the literature.^15,48^Fig. 17 illustrates the dilatometric analysis of the commercial β-TCP (S_0_) and synthesized S_10_ samples. Both specimens displayed a minor linear shrinkage of approximately 0.25% from room temperature up to 200 °C, which is attributed to the desorption of physically bound water. Within the 200–950 °C temperature interval, a continuous thermal expansion (dilatation) of roughly 0.7% occurred, followed by the onset of sintering above 1000 °C. The commercial β-TCP reached a total shrinkage of about 2.5% at 1200 °C. In contrast, the S_10_ sample exhibited an initial shrinkage of 1.5% at 1150 °C, followed by a distinct plateau or delay in densification before undergoing further shrinkage above 1300 °C to a maximum value of approximately 3.5%. This divergence in high-temperature dimensional behavior can likely be attributed to variations in the particle size distribution and granulometry of the starting powders, as previously indicated in Fig. 13. A similar dilatometric behavior was documented by Bouslama *et al.*,^49^ who reported a comparable delay in the sintering onset temperature to 1180 °C. Conversely, Ghosh and Sarkar^50^ observed that β-TCP densification proceeded rapidly within the single-phase β-TCP stability regime, whereas the nucleation of the secondary α-TCP above 1250 °C induced a characteristic volume expansion. This expansion mechanism contrasts with the continuous shrinkage behavior observed in the present study. Similarly, Destainville *et al.*^51^ noted thermal expansion up to 750 °C followed by a maximum densification rate between 950 °C and 1000 °C, culminating in a significant expansion at 1200 °C driven by the polymorphic β to α transformation. These conflicting literature profiles highlight how the choice of chemical synthesis pathway directly dictates the subsequent thermal and sintering kinetics of β to α transformation compacts.^50^

## Conclusion

This study highlights a sustainable approach offering a dual benefit, based on the recovery of dental plaster waste as tricalcium β-phosphate. It is important to note that this research aims to address the current lack of specific legislation regarding the disposal of dental plaster models in Tunisia. Using a Box–Behnken experimental design and response surface methodology, we identified the predicted optimal synthesis parameters: a solid-to-liquid ratio of 3.7, a heating temperature of 1150 °C and a heating time of 2.2 hours. Experiment S_10_ is close to this point, furthermore, it exhibits higher crystallinity than the reference sample (commercial β-TCP). It is therefore considered to be our experimental optimum and was subsequently subjected to further characterization in order to compare its structural and compositional comparability with commercial tricalcium phosphate. The XRD patterns exhibited sharp, well-defined peaks corresponding exclusively to the β-TCP phase, indicating high crystallinity. Concurrently, FTIR analysis confirmed the total absence of organic or chemical impurities. Furthermore, EDX analysis revealed a Ca/P molar ratio of 1.48, which closely aligns with the theoretical value and corroborates the successful synthesis of β-tricalcium phosphate. Dilatometric analysis revealed slight behavioral variations compared to commercial standards.

This variation is likely due to differences in particle size. In conclusion, recovering dental plaster waste for β-tricalcium phosphate synthesis provides a practical approach to material reuse. By connecting waste management with the production of dental laboratory supplies, this research offers a useful model for reducing waste within the field. Future work will focus on *in vitro* biocompatibility assays to evaluate the material's suitability for bone tissue engineering applications.

## Author contributions

Ftiti Sondes: investigation, writing – original draft. Tounsi Hassib, Samet Basma, Guidara Awatef and Cifuentes Sandra: formal analysis, methodology, validation. Tounsi Hassib, Samet Basma, Guidara Awatef and Cifuentes Sandra: validation, conceptualization, supervision.

## Conflicts of interest

The authors declare no conflict of interest.

## Data Availability

The authors confirm that the data supporting the findings of this study are available within the article.
